# Cystatin C, a novel indicator of renal function, reflects severity of cerebral microbleeds

**DOI:** 10.1186/1471-2377-14-127

**Published:** 2014-06-12

**Authors:** Mi-Young Oh, Hyon Lee, Joon Soon Kim, Wi-Sun Ryu, Seung-Hoon Lee, Sang-Bae Ko, Chulho Kim, Chang Hun Kim, Byung-Woo Yoon

**Affiliations:** 1 Department of Neurology, Seoul National University Hospital, 101 Daehang-ro, Jongno-gu, Seoul, Republic of Korea; 2 Department of Neurology, Dogguk University Ilsan Hospital, Goyang, Republic of Korea; 3 Department of Neurology, Chuncheon Sacred Heart Hospital, Hallym University College of Medicine, Chuncheon, Republic of Korea

**Keywords:** Cystatin C, Estimated glomerular filtration rate, Microalbuminuria, Cerebral microbleeds

## Abstract

**Background:**

Chronic renal insufficiency, diagnosed using creatinine based estimated glomerular filtration rate (GFR) or microalbumiuria, has been associated with the presence of cerebral microbleeds (CMBs). Cystatin C has been shown to be a more sensitive renal indicator than conventional renal markers. Under the assumption that similar pathologic mechanisms of the small vessel exist in the brain and kidney, we hypothesized that the levels of cystatin C may delineate the relationship between CMBs and renal insufficiency by detecting subclinical kidney dysfunction, which may be underestimated by other indicators, and thus reflect the severity of CMBs more accurately.

**Methods:**

Data was prospectively collected for 683 patients with ischemic stroke. The severity of CMBs was categorized by the number of lesions. Patients were divided into quartiles of cystatin C, estimated GFR and microalbumin/creatinine ratios. Ordinal logistic regression analysis was used to examine the association of each renal indicator with CMBs.

**Results:**

In models including both quartiles of cystatin C and estimated GFR, only cystatin C quartiles were significant (the highest vs. the lowest, adjusted OR, 1.88; 95% CI 1.05-3.38; *p* = 0.03) in contrast to estimated GFR (the highest vs. the lowest, adjusted OR, 1.28; 95% CI 0.38-4.36; *p* = 0.70). A model including both quartiles of cystatin C and microalbumin/creatinine ratio also showed that only cystatin C quartiles was associated with CMBs (the highest vs. the lowest, adjusted OR, 2.06; 95% CI 1.07-3.94; *p* = 0.03). These associations were also observed in the logistic models using log transformed-cystatin C, albumin/creatinine ratio and estimated GFR as continuous variables. Cystatin C was a significant indicator of deep or infratenorial CMBs, but not strictly lobar CMBs. In addition, cystatin C showed the greatest significance in c-statistics for the presence of CMBs (AUC = 0.73 ± 0.03; 95% CI 0.66-0.76; *p* = 0.02).

**Conclusion:**

Cystatin C may be the most sensitive indicator of CMB severity among the renal disease markers.

## Background

Cerebral microbleeds (CMBs) are discrete or isolated, punctate, hypo-intense lesions, smaller than 5 mm on T2- weighted MRI [1,2]. They are clinically silent but are strongly associated with advanced microvascular ischemic disease and are a marker for increased risk of future intracranial hemorrhagic events [3,4]. CMBs are a reflection of generalized microangiopathy in cerebral small vessel disease [5,6]. Small vessel disease in the brain and kidneys are closely related through anatomical and vasoregulatory similarities, including high perfusion pressure, low vascular resistance and the critical role of nitric oxide in maintaining the microcirculation of the glomerulus or cerebral perforating arterioles [7]. Recent studies also reported that a close relationship exists between renal dysfunction and the presence of CMBs in stroke patients [8,9]. With this context in mind, an appropriate indicator may exist that reflects the severity of the small vessel pathologies of both the brain and the kidneys. Most of the previous studies defined renal insufficiency based on the conventional, creatinine-based renal indicators, estimated glomerular filtration rate (GFR) or microalbuminuria [10,11]. Cystatin C, a cysteine proteinase inhibitor, has been proposed to be an alternative marker for renal function. It is abundant in the serum and less dependent on extra renal factors compared to creatinine [12,13]. It also has a greater sensitivity for revealing mild renal dysfunction even in presumably healthy individuals compared to conventional renal indicators [14,15]. In addition, cystatin C levels have been shown to correlate with silent cerebral infarctions and white matter lesions [16,17]. However the association between the cystatin C and CMB severity has not been reported. We examined whether cystatin C is more strongly associated with CMBs than the estimated GFR or microalbumin/creatinine ratio.

## Methods

### Patients

Data was prospectively collected for 714 consecutive Korean patients who were admitted to a tertiary medical center with an ischemic stroke within 7 days of onset between January 2008 and May 2011. The enrolled population was ethnically homogeneous. We excluded patients whose GRE MR images were not suitable for evaluating CMBs as a result of motion or susceptibility artifacts (n = 31). A total of 683 patients were included for analysis. This study was approved by Seoul National University Hospital institutional review board.

### Laboratory and clinical information

Serum cystatin C was measured from blood samples drawn upon admission with the use of a BN II nephelometer (Siemens Healthcare Diagnostics, Inc) with a particle-enhanced immunonephelometric assay (N Latex cystatin C, Siemens Healthcare Diagnostics, Inc) [18]. *S*erum creatinine was measured by the rate Jaffe method [19]. The intra-individual coefficient of variation was approximately 2%. The estimated GFR was calculated by the abbreviated (4-variable) Modification of Diet and Renal Disease Study formula as follows: for creatinine in umol/L, estimated GFR = (serum creatinine/88.4)^–1.154^ x (age^–0.203^) x (0.742, if female) [20]. The urine microalbumin/creatinine ratio was measured by a single spot sample at admission using nephelometry and was reported as micrograms of albumin per milligram creatinine [21]. All patients had serum panels including hemoglobin A1c, lipid parameters, and hemoglobin drawn early in the morning after fasting overnight. We collected baseline demographic and clinical information for all study participants, including age, sex, use of antithrombotic or anticoagulant medication and presence of cardiovascular risk factors such as hypertension (determined by the previous use of antihypertensive medication, a systolic blood pressure > 140 mm Hg or a diastolic blood pressure > 90 mm Hg at discharge), diabetes (determined by the previous use of antidiabetic medication, a fasting blood glucose > 7.0 mmol/L or a postprandial blood glucose after 2 hours >11.1 mmol/L at discharge), dyslipidemia (determined by the previous use of lipid-lowering medication or a total cholesterol > 6.2 mmol/L), heart disease (defined as patients with previously diagnosed atrial fibrillation, myocardial infarction, valvular heart disease or cardiomyopathy), and previous stroke. We classified the stroke mechanism of the patients based on the Trial of ORG 10172 in Acute Stroke Treatment (TOAST) criteria [22].

### Imaging information

MRI was performed using a 1.5 T superconducting magnet system (GE Medical System, Milwaukee, WI). Gradient Echo (GRE) T2*-weighted magnetic resonance imaging (MRI) was performed as part of a routine protocol, and the images were obtained in the axial plane with the following parameters: repetition time/echo time = 500/15 msec; flip angle = 26°; matrix size = 256 _ 192; slice thickness = 6 mm; and gap width = 2 mm. Standard T2-weighted and fluid attenuated inversion recovery (FLAIR) sequences were also obtained. CMBs were defined as a well-defined focal area of low signal on the GRE MRI less than 10 mm in diameter, and they were counted throughout the whole brain by the two trained neurologists. All GRE scans of every patient were performed upon diagnosis of the ischemic stroke and were examined twice for intra-rater variability (*k* = 0.93, *p* < 0.01). The interval duration was four weeks. The CMB grades of two raters were compared to each other to determine inter-rater variability (*k* = 0.95, *p* < 0.01) [23]. CMB mimicking lesions (e.g. vessels, mineralization, air-bone interfaces and partial volume artifacts at the edges of the cerebellum) were excluded. The lesions were stratified by location: the corticosubcortical area (cortex, subcortex and white matter), deep gray mater (basal ganglia and thalamus) and infratentorial area (brain stem and cerebellum) [24]. We divided the CMBs into strictly lobar and deep or infratentorial (with or without lobar CMBs) groups according to location. Persons who had more than one CMB restricted to the lobar location were classified as “strictly lobar CMBs” patients. CMBs in a deep or infratentorial location with or without lobar CMBs were classified as “deep or infratentorial CMBs” [25]. We graded the severity of the CMBs based on the total number of CMBs: normal = no CMBs; mild = 1 to 4; moderate = 5 to 9; and severe ≧ 10 [24]. White matter lesions seen on T2*-weighted or FLAIR images were defined according to the criteria of Fazekas et al.: normal, being absent of white matter lesions; punctuate, showing multiple periventricular hyperintense punctate lesions; early confluent, showing early confluence of lesion foci; and confluent, showing confluence of multiple areas [26].

### Statistical analysis

The distributions of demographic, clinical, laboratory and stroke data according to cystatin C levels were determined using the chi-square test for trends in proportion or by one-way analysis of variance with the Bonferroni method for post hoc analysis and the Kruskal-Wallis test as appropriate. We used the chi square test for trend to compare the distributions of grades of CMBs according to the quartiles of cystatin C. Multi-colinearity between the variables were assessed by the Pearson correlation analysis (cystatin C and estimated GFR, *r* = 0.43, *p* < 0.01; cystatin C and microalbumin/creatinine ratio, *r* = 0.23, *p* < 0.01; cystatin C and creatinine, *r* = 0.52, *p* < 0.01; estimated GFR and microalbumin/creatinine, *r* = 0.81, *p* < 0.01).

To examine the association between CMB grades and cystatin C according to groups with lacunar or non lacunar stroke, we conducted partial correlation analyses, adjusted for age and sex. We compared each correlation coeffient using Fisher’s Z transformation. Proportional odds models for logistic regression analyses were performed on the quartiles of cystain C, microalbumin/creatinine ratio and estimated GFR. First, the logistic model was conducted using the quartiles of cystatin C, estimated GFR or microalbumin/creatinine ratio separately. The second model included two of the three indicators. Among the three renal indicators, the multicolinearlity of the estimated GFR and the microalbumin/creatine ratio was 0.81, more than 0.67 which may lead to spurious results in logistic regression analysis [27]. Thus, we analyzed the estimated GFR and microalbumin/creatine using the individual logistic regression analysis with cystatin C. The third ordinal logistic regression analyses were also performed on the standard deviation of the log-transformed values of cystatin C or estimated GFR and microalbumin/creatinine ratio. We also conducted the fourth ordinal logistic regression analyses according to the location of CMBs (strictly vs. deep or infratenrorial CMBs).

These logistic models were adjusted for clinical confounders including age, gender, total cholesterol, diabetes, hypertension, dyslipidemia, previous heart disease, smoking, previous anti thrombotic or anticoagulant use, and white matter lesions. The proportional odds assumption was assessed by the parallel line test. Significance was set at a 2-tailed *p* < 0.05 level. We assessed the improvement in discrimination by comparing the area under the receiver operator characteristic curves (AUC) in models with and without each renal indicator. The ROC curve was generated to compare the relationship between each renal indicator and the presence of CMBs using multivariable logistic regression that included variables of age, gender, diabetes, dyslipidemia, hypertension, smoking, total cholesterol, anti thrombotic or anticoagulant use, and white matter lesion volume with or without cystatin C, microalbumiuria, and estimated GFR. We presented the values in frequencies (percentages), means ± SDs, or medians (interquartile ranges (IQR)) as appropriate. All the statistical analyses were performed using SPSS 17.0.1(SPSS Inc, Chicago, IL) and STATA version 7.0 (STATA Corp, College Station, Tex).

## Results

### Baseline characteristics

Among a total of 683 stroke patients, 443 (63.4%) were male. The average age was 66.6 ± 12.3 years (range of 21 to 96 years). The number of patients with small vessel occlusions was 145 (21.2%), and the number of patients with large artery thrombosis was 196 (28.7%). Cardioembolism was determined as the cause of stroke in 136 (19.9%) patients, 96 (14.1%) patients were classified as having a stroke of other determined etiology according to the TOAST classification, and 110 (16.1%) patients had a stroke of unknown etiology. Three hundred and forty-three patients had hypertension (50.2%), 181 patients (26.5%) had diabetes, 200 patients (29.3%) had dyslipidemia, and 151 patients (22.1%) had a history of heart disease. Eighty eight patients were taking Aspirin (ATC classification, B01AC06). Eight patients were prescribed other anti platelet medications including clopidogrel (B01AC04), ticlopidine (B01AC05), and cilostazole (B01AC23). Thirty-four patients were on warfarin (BO1AA03).

The cystatin C concentrations ranged from 27.0 to 603.7 nmol/L (median = 54.7, inter quartile range [IQR] = 47.2 to 66.7). The estimated GFR ranged from 3.5 to 249.5 mL/min/1.73 m^2^ (mean ± SD = 80.5 ± 26.0). The albumin/creatinine ratio was from 3.0 to 190.7 μg/mg (median = 24.0; IQR = 11.0, 100.75). The subjects with higher grades of CMBs were more likely to have a previous history of stroke, higher cystatin C levels and more confluent white matter lesions. Initial NIHSS scores or history of antithrombotic medication use were not different among the groups (Table 1). Fifty-four patients were labeled as “strictly lobar CMBs” patients, and 135 patients were classified as the “deep or infratentorial CMBs” group.

**Table 1:** Baseline profiles

**Variables**		**Grade**			
	**Normal**	**Mild**	**Moderate**	**Severe**	***P***
No. of CMBs	0	1-4	5-9	≧10	
No. of Cases, n (%)	494(72.3)	141(20.6)	24(3.5)	24(3.5)	
Demographic data					
Age, mean ± SD	65.7 ± 12.4	69.1 ± 10.9	67.2 ± 13.8	66.6 ± 12.2	0.02^†^
Male, n (%)	311(63.0)	90(63.8)	15(62.5)	17(70.8)	0.89^**^
Risk factors, n (%)					
Hypertension	229(46.4)	85(60.3)	14(58.3)	15(62.5)	0.01^**^
Diabetes	125(25.3)	46(32.6)	4(16.7)	6(25.0)	0.23^**^
Dyslipidemia	152(30.8)	40(28.4)	5(20.8)	3(12.5)	0.20^**^
Smoking	181(36.6)	43(30.5)	3(12.5)	5(20.8)	0.08^**^
History of heart disease	105(21.3)	37(26.2)	4(16.7)	5(20.8)	0.56^**^
History of previous stroke	83(16.8)	39(27.7)	9(37.5)	8(33.3)	<0.01^**^
Initial NIHSS^*^ score	3[1,7]	3[1,5]	4[2,6]	3[1,5]	0.63^‡^
Stroke information, n (%)					
Small vessel occlusion	88(17.8)	37(26.2)	11(45.8)	9(37.5)	<0.02
Large artery thrombosis	153(31.0)	35(24.8)	3(12.5)	5(20.8)	
Cardio embolism	87(19.6)	33(23.4)	3(12.5)	3(12.5)	
Cryptogenic	79(16.0)	21(14.9)	5(20.8)	5(20.8)	
Other etiology	79(15.6)	15(10.6)	2(8.3)	2(8.3)	
Laboratory data					
Hemoglobin (g/dL)	13.6 ± 2.0	13.3 ± 2.0	13.8 ± 2.1	18.6 ± 5.1	<0.01^†^
Total Cholesterol (mmol/L)	4.5 ± 1.1	4.3 ± 1.1	4.6 ± 0.8	4.6 ± 0.7	0.24^†^
White blood cell count (10^6^/μL)	7.8 ± 2.8	7.7 ± 3.0	7.3 ± 2.0	6.8 ± 2.1	0.28
Cystatin C,	53.9	60.7	61.0	64.7	0.01^‡^
median[IQR] (nmol/L)	[46.4,63.7]	[47.9,74.9]	[51.9,68.9]	[54.9,82.2]	
Creatinine (μmol/L)	99.3 ± 69.1	114.5 ± 83.7	92.3 ± 20.3	148.0 ± 52.9	0.01^†^
Estimated GFR^*^,	82.2 ± 25.7	74.1 ± 26.4	80.8 ± 26.5	75.2 ± 25.4	<0.01^†^
mean ± SD (ml/min/1.73 m^2^)					
Microalbumin/Creatinine,	0.02	0.03	0.01	0.03	0.21^‡^
median[IQR] (μg/mg)	[0.01,0.08]	[0.01,0.18]	[0.01,0.10]	[0.01,0.20]	
Severity of WML^*^, n (%)					
Normal	207(42.2)	33(23.6)	1(4.2)	0(0)	<0.01^**^
Punctate	131(26.7)	34(24.3)	1(4.2)	0(0)	
Early confluent	46(9.4)	21(15.0)	3(12.5)	2(8.3)	
Confluent	107(21.8)	52(37.1)	19(79.2)	22(11.0)	
Anti thrombotics, n (%)	57(11.5)	28(19.9)	6(25.0)	5(20.8)	0.91^**^
Aspirin	49(9.9)	28(19.9)	6(25.0)	5920.8)	
Other anti thrombotics	8(1.6)	0	0	0	
Anticoagulant, warfarin, n (%)	18(3.6)	11(7.8)	3(12.5)	2(8.3)	0.31^**^

*NIHSS; National Institute of Health Stroke Scale, GFR;glomerular filtration rate, WML; white matter lesions.

*p*-values were calculated by the Chi-square test^**^, one-way analysis of variance with Bonferroni method^†^and Kruskal-Wallis test^‡^.

Interestingly, the association between the CMB grades and cystatin C in the patients with lacunar stroke showed a stronger correlation than that in the patients with non lacunar stroke. (*r* = 0.26 at *p* < 0.01 vs. *r* = 0.09 at *p* < 0.05; Z score = 1.72 at *p* = 0.04).

### The association between cystatin C, estimated GFR and CMBs

Patients were divided into four groups based on the quartile values of serum cystatin C (Q1 < 47.2, Q2 = 47.2-54.7, Q3 = 54.7-66.7, and Q4 > 66.7 nmol/L), or the quartiles of estimated GFR (Q4 > 66.1, Q3 = 66.1-78.8, Q2 = 78.8-94.7, and Q1 > 94.7 ml/min/1.73 m^2^). The proportion of patients with a moderate to severe number of CMBs gradually increased as the cystatin C levels increased (*p* for trend <0.01).

Estimated GFR and cystatin C levels were associated with CMBs using an independent logistic model. Compared to patients with the lowest levels of cystatin C, patients with high cystatin C had a more significant association with the presence of CMBs. After adjusting for confounders, the cystatin C levels (adjusted OR, 1.90) remained significant in individual models. The estimated GFR analysis failed to show significance (adjusted OR 1.82). In a second model including both estimated GFR and cystatin C levels, cystatin C was independently associated with an increased risk of CMBs. Those with the highest cystatin C levels had a 1.88 fold higher risk of severe CMBs (95% confidential interval [CI], 1.05-3.38; *p* = 0.03). The estimated GFR failed to show a significant association with CMB severity (adjusted OR, 1.28; 95% CI, 0.38-4.36; *p* = 0.70). Age, hypertension, smoking history and white matter lesion volume showed a significant association with CMB severity after adjusting for confounders (Table 2). After using clinical categories of estimated GFR based on chronic kidney disease stage, only cystatin C remained significantly associated with the CMBs (see Additional file 1). These results were reproduced (see Additional file 2) after excluding patients with renal failure (n = 13). Further, we conducted analyses using the newly developed estimated GFR equation based on both creatinine and cystatin C, and found that the estimated GFR using cystatin C or based on both creatinine and cystatin C were better predictors of CMB severity than the estimated GFR based creatinine alone. (see Additional file 3) [28].

**Table 2:** Ordinal logistic regression analysis for the association of renal indicators and cerebral microbleeds

					**Model1**^**†**^			**Model2**^**‡**^					
**Variables**	**N**	**Unadjusted OR**	**95% CI**	***P***	**Adjusted OR**	**95% CI**	***p***	**Adjusted OR**	**95% CI**	***p***	**Adjusted OR**	**95% CI**	***p***
Age		1.02	1.01-1.04	<0.01	1.02	1.02-1.04	<0.01	1.01	1.00-1.03	0.07	1.02	1.01-1.04	0.02
Male		1.08	0.65-1.31	0.66	1.36	0.73-1.36	0.76	1.36	0.91-2.03	0.13	1.31	0.87-1.31	0.20
Hypertension		1.74	1.24-2.44	<0.01	1.43	1.05-2.18	0.03	1.51	1.05-2.19	0.03	1.53	1.06-2.23	0.02
Diabetes		1.19	0.82-1.72	0.36	0.85	0.57-1.28	0.45	1.23	0.74-1.68	0.59	1.18	0.78-1.78	0.44
Dyslipidemia		1.35	0.51-1.08	0.12	0.68	0.45-1.02	0.06	1.22	1.00-2.19	0.05	1.49	1.00-2.22	0.05
Heart disease		1.15	0.70-1.70	0.48	1.05	0.66-1.66	0.82	1.05	0.70-1.60	0.80	1.00	0.65-1.52	1.00
Smoking		1.62	1.12-2.34	0.01	1.35	0.86-2.13	0.05	1.91	1.25-2.92	<0.01	1.52	1.01-2.28	<0.01
Anticoagulant or thrombotics		2.26	1.53-2,26	<0.01	1.72	1.08-2.72	0.02	1.51	1.00-2.29	0.05	1.48	0.98-2.25	0.07
Total cholesterol		1.07	0.91-1.26	0.42	1.01	0.81-1.01	0.43	1.01	0.85-1.21	0.89	1.03	0.87-1.23	0.72
WML*													
Confluent		6.24	3.96-9.83	<0.01	4.08	2.37-7.02	<0.01	4.82	2.98-7.80	<0.01	4.56	2.80-7.41	<0.01
Early confluent		3.92	2.47-6.22	<0.01	2.66	1.60-4.41	<0.01	2.72	1.74-4.25	<0.01	2.62	1.67-4.12	<0.01
Puctate		1.82	1.06-3.14	0.03	1.31	0.72-2.39	0.38	1.57	0.91-2.70	0.11	1.45	0.83-2.51	0.19
Normal		ref			ref						ref		
*p* for trend				<0.01			<0.01						<0.01
Quartiles of cystatin C													
Q4(≥66.7)	168	2.19	1.40-3.40	<0.01	1.90	1.07-3.37	0.02				1.88	1.05-3.38	0.03
Q3(54.7-66.7)	173	1.57	0.92-2.46	0.10	1.31	0.45-1.28	0.35				1.24	0.74-2.08	0.42
Q2(47.2-54.7)	162	0.98	0.61-1.58	0.16	1.54	0.85-2.80	0.16				0.83	0.49-1.41	0.49
Q1(≤47.2), ref	180												
*p* for trend				<0.01			<0.01						<0.01
Quartiles of eGFR*													
Q4(≤66.1)	171	2.26	1.42-3.62	<0.01				1.82	0.38-4.00	0.72	1.28	0.38-4.36	0.70
Q3(66.1-78.8)	175	1.23	0.50-1.33	0.41				1.44	0.95-3.99	0.67	0.78	0.35-1.70	0.93
Q2(78.8-94.7)	164	1.18	0.50-1.40	0.51				1.33	0.70-2.11	0.50	1.11	0.61-2.03	1.11
Q1(≥94.7),ref	173												
*p* for trend				<0.01						<0.01			0.51

*WML; white matter lesions, eGFR; estimated glomerular filtration rate.

†Model 1: quartiles of estimated GFR and cystatin C were analyzed separately in the individual ordinal logistic regression model.

‡Model 2: quartiles of estimated GFR and cystatin C were analyzed together in the same logistic regression model.

Model 1 and model 2 were adjusted for covariates; age, sex, total cholesterol, diabetes, hypertension, dyslipidemia, previous heart disease, smoking, previous anti thrombotic or anticoagulant use, and white matter lesions.

### The association between cystatin C, microalbuminuria and CMBs

We also used additional multivariable models to examine the association between cystatin C, microalbumin/creatinine ratio and CMBs. Crude and adjusted ORs for the severity of CMBs are presented in Table 3. The higher quartiles of microalbuminuria were not associated with CMBs, as compared to the lowest quartile. The crude ORs of the microalbumin/creatinine ratios showed an increasing, but statistically insignificant trend (ORs of Q4, Q3, Q2, 1.34, 1.28, 0.86, respectively; *p* for trend = 0.20). After adjusting for other variables, cystatin C (OR, 2.06; 95% CI, 1.07-3.94; *p* = 0.03) remained as an independent predictor of the severity of CMBs, in contrast to microalbumiuria levels (OR, 1.34, 95% CI, 0.71-1.43, *p* = 0.36).

**Table 3:** Ordinal logistic regression analysis for the association of renal indicators and cerebral microbleeds

					**Model1**^**†**^			**Model2**^**‡**^					
**Variables**	**N**	**Unadjusted OR**	**95% CI**	***P***	**Adjusted OR**	**95% CI**	***p***	**Adjusted OR**	**95% CI**	***p***	**Adjusted OR**	**95% CI**	***p***
Age, per year		1.02	1.01-1.04	<0.01	1.02	1.02-1.04	<0.01	1.02	1.00-1.04	0.06	1.01	1.01-1.05	0.01
Male		1.08	0.65-1.31	0.66	1.36	0.73-1.36	0.76	1.39	0.87-2.22	0.17	1.31	0.86-2.12	0.27
Hypertension		1.74	1.24-2.44	<0.01	1.43	1.05-2.18	0.03	1.77	1.14-2.78	<0.01	1.83	1.17-2.86	<0.01
Diabetes		1.19	0.82-1.72	0.36	0.85	0.57-1.28	0.45	1.04	0.64-1.68	0.88	1.10	0.68-1.79	0.70
Dyslipidemia		1.35	0.51-1.08	0.12	0.68	0.45-1.02	0.06	1.72	1.08-2.73	0.02	1.76	1.10-2.82	0.02
Heart disease		1.15	0.70-1.70	0.48	1.05	0.66-1.66	0.82	1.03	0.63-1.67	0.91	1.06	0.65-1.74	0.81
Smoking		1.62	1.12-2.34	0.01	1.35	0.86-2.13	0.05	1.22	0.76-1.96	0.41	1.26	0.78-2.04	0.35
Anticoagulant or thrombotics		2.26	1.53-2,26	<0.01	1.72	1.08-2.72	0.02	1.67	1.01-2.76	0.05	1.63	0.98-2.70	0.26
Total cholesterol, per mmol/L		1.07	0.91-1.26	0.42	1.01	0.81-1.01	0.43	1.01	0.83-1.23	0.93	1.05	0.86-1.28	0.27
WML*													
Confluent		6.24	3.96-9.83	<0.01	4.08	2.37-7.02	<0.01	4.87	2.78-8.52	<0.01	4.56	2.59-8.03	<0.01
Early confluent		3.92	2.47-6.22	<0.01	2.66	1.60-4.41	<0.01	2.67	1.57-4.53	<0.01	2.54	1.49-4.34	<0.01
Puctate		1.82	1.06-3.14	0.03	1.31	0.72-2.39	0.38	1.60	0.85-3.01	0.15	.1.49	0.79-2.83	0.22
Normal,ref													
*p* for trend				<0.01						<0.01			<0.01
Quartiles of cystatin C													
Q4(≥66.7)	168	2.19	1.40-3.40	<0.01	1.90	1.07-3.37	0.02				2.06	1.07-3.94	0.03
Q3(54.7-66.7)	173	1.57	0.92-2.46	0.10	1.31	0.45-1.28	0.35				2.20	1.18-4.09	0.01
Q2(47.2-54.7)	162	0.98	0.61-1.58	0.16	1.54	0.85-2.80	0.16				1.36	0.78-2.36	0.27
Q1(≤47.2), ref	180												
*p* for trend				<0.01			<0.01						<0.01
Quartiles of Alb/Cr*,													
Q4(≥100.8)	160	1.52	0.87-2.66	0.14				1.61	0.88-2.95	0.13	1.34	0.71-1.43	0.36
Q3(24.0-.100.8)	136	1.20	0.70-2.06	0.51				1.32	0.75-2.29	0.33	1.28	0.73-1.48	0.38
Q2(11.0-24.0)	217	0.97	0.52-1.80	0.91				1.09	0.59-2.03	0.78	0.86	0.53-1.61	0.76
Q1(≤11.0),ref	170												
*p* for trend				0.10						0.16			0.20

*WML;white matter lesions, Alb/Cr; albumin/creatinine ratio.

†Model 1: quartiles of microalbumin/creatinine and cystatin C were analyzed separately in the individual ordinal logistic regression model.

‡Model 2: quartiles of microalbumin/creatinine and cystatin C were analyzed together in the same logistic regression model.

Model 1 and model 2 were adjusted for covariates; age, sex, total cholesterol, diabetes, hypertension, dyslipidemia, previous heart disease, smoking, previous anti thrombotic or anticoagulant use, and white matter lesions.

In Table 4, log transformed cystatin C levels showed an association with CMBs, but other renal indicators did not. These analyses were repeated (see Additional file 4) excluding the patients with renal failure (n = 13).

**Table 4:** logistic regression analysis for the association of renal indicators and cerebral microbleeds

				**Model 1**^**†**^			**Model 2**^**‡**^					
**Variables**	**Unadjusted OR**	**95% CI**	***p***	**Adjusted OR**	**95% CI**	***p***	**Adjusted OR**	**95% CI**	***p***	**Adjusted OR**		***p***
Log-Cystatin C, per SD^*^	1.37	1.18-1.59	<0.01	1.30	1.05-1.69	0.02	1.27	1.04-1.61	0.02	1.73	1.82-3.08	<0.01
Log-albumin/Creatinine, per SD	0.90	0.81-1.01	0.08	0.90	0.82-1.03	0.93				1.00	0.87-1.14	0.96
Estimated GFR^*^, per SD	1.01	0.64-0.91	<0.01	1.01	0.82-1.30	0.80	1.01	0.80-1.27	0.86			

^*^SD; standard deviation, GFR; glomerular filtration rate.

^†^Model 1: Estimated GFR and log transformed cystatin C, and microalbumin/creatinine ratios were analyzed separately in an individual ordinal logistic model.

^‡^Model 2: the first ordinal logistic regression analysis was performed using log transformed value of cystatin C and estimated GFR together. The second model used log transformed values of cystatin C and microalbumin/creatinine ratios as variables. All models were adjusted for covariates: age, sex, total cholesterol, diabetes, hypertension, dyslipidemia, previous heart disease, smoking, previous anti thrombotic or anticoagulant use, and white matter lesions.

### The association between cystatin C and the location of CMBs

When we divided the CMB patients into strictly lobar and deep or infratentorial (with or without lobar CMBs) groups, the association between cystatin C quartiles and CMBs was significant for the deep or infratentorial CMB group but not for the strictly lobar CMB group (Table 5). The adjusted OR of the fourth quartile of cystatin C for the deep of infratentorial CMB grades was 6.67 (95% CI, 1.48–2.64; *p* = 0.01). In addition, only cystatin C remained a significant indicator of CMBs grade in the deep or infratentorial CMB group among the three indicators (see Additional file 5 and Additional file 6).

**Table 5:** Ordinal logistic regression analyses according to the location of CMBs

**Variables**	**N**	**Unadjusted OR**	**95% CI**	***P***	**Adjusted OR**	**95% CI**	***P***
Strictly lobar CMBs	54						
Quartiles of cystatin C							
Q4(≥66.7)	23	6.14	0.01-1.78	0.13	5.23	0.30-8.98	0.13
Q3(54.7-66.7)	14	4.84	0.01-3.80	0.29	2.82	0.46-1.69	0.11
Q2(47.2-54.7)	5	1.54	0.03-11.2	0.76	1.37	0.05-3.77	0.85
Q1(≤47.2), ref	12						
*p* for trend				0.10			0.50
Deep or infratentorial CMBs	135						
Quartiles of cystatin C							
Q4(≥66.7)	45	5.00	1.30-1.91	0.02	6.27	1.48-2.64	0.01
Q3(54.7-66.7)	39	1.50	0.51-4.34	0.46	2.48	0.76-8.02	0.13
Q2(47.2-54.7)	23	1.00	0.40-2.28	0.93	1.10	0.43-2.78	0.83
Q1(≤47.2),ref	28						
*p* for trend				0.02			0.02

Adjusted for the covariates: age, sex, total cholesterol, diabetes, hypertension, dyslipidemia, previous heart disease, smoking, previous anti thrombotic or anticoagulant use and white matter lesions.

### The predictive value of cystatin C, microalbuminuria, and estimated GFR for CMBs

The AUC of the logistic regression model calculated without any renal indicators was 0.66 ± 0.03 (95% CI, 0.59-0.70; *p* = 0.02). The addition of cystatin C increased the AUC (0.73 ± 0.03; 95% CI, 0.66-0.76; *p* = 0.02; difference = 0.07; 95% CI, 0.01-0.12; *p* < 0.01).

**Figure 1:**
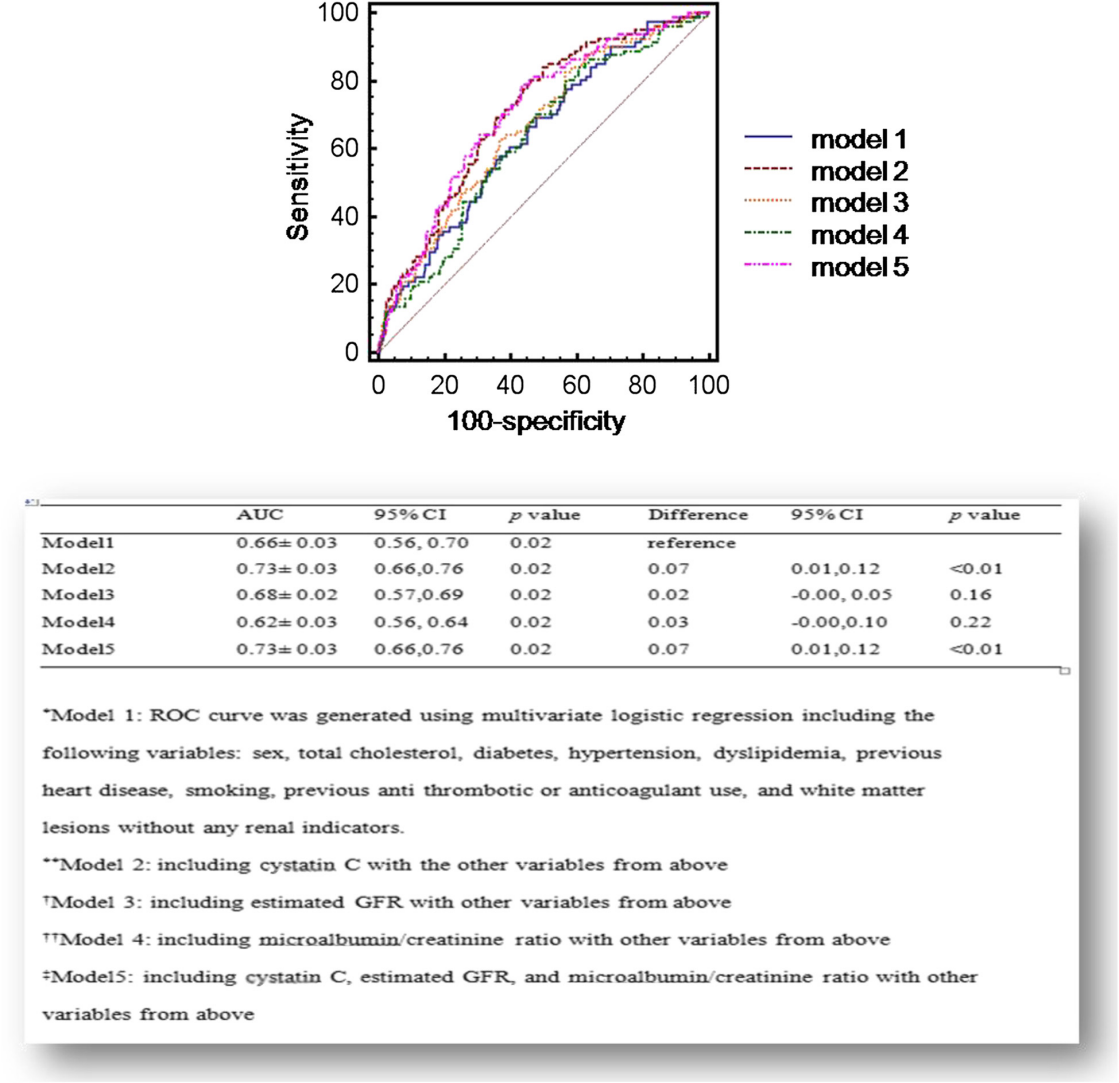
The predictive value of each renal indicator for the presence of CMBs.

In contrast, adding the estimated GFR (0.68 ± 0.02; 95% CI, 0.57-0.69; *p* = 0.02) or microalbumiuria (0.62 ± 0.03; 95% CI, 0.56-0.64; *p* = 0.02) did not show a profound incremental change compared to the model without the indicators. The AUC of the logistic regression model including all the indicators did not show a difference between that of the model adding the cystatin C alone (difference < 0.01; 95% CI, 0.00-0.02; *p* = 0.86). Taken together, the levels of cystatin C appear to have the greatest discriminating power among the three indictors (Figure 1).

## Discussion

We found that higher cystatin C concentrations showed a greater association with severe CMB pathology especially in patients of the highest quartile. One standard deviation increase in the log-transformation of cystatin C levels also showed a closer relationship with severe CMBs. The estimated GFR and microalbumin/creatinine ratio failed to show a significant association with the number of CMBs. Moreover, the association was sustained in patients with deep or infratentorial CMBs but not among those with strictly lobar CMBs. In addition, the correlation between the CMB grades and cystatin C in patients with lacunar stroke showed a stronger association than that in patients with non lacunar stroke. Cystatin C was the most powerful indicator for CMBs among the three renal markers. Our results corroborate previous studies showing that renal dysfunction is independently associated with CMBs [8,29]. Furthermore, we showed that cystatin C may reflect the severity of CMBs in a dose dependent manner more accurately than other renal indicators.

Cystatin C concentration is less affected by extrarenal factors compared to creatinine [12]. It has been used as a predictor of coronary heart disease, heart failure, and all causes of mortality in various populations by the detection of subclinical renal insufficiency [30-32]. Its prognostic value for adverse outcomes was independent of conventional renal parameters [31-33]. In studies of small arteriopathies, cystatin C also showed a strong association with white mater lesions or silent brain infarctions compared to estimated GFR [16,17]. Our results seem to support the finding of these previous studies.

White matter lesions were significantly associated with CMBs in this study. Recent studies suggested CMBs may be one of pathologic continuum with white matter lesions and lacunar infarcts [34]. Spontaneous hypertensive rat model reported that the initial early, silent damage to the blood brain barrier initiates a pathological cascade-via microbleeds- that leads to the formation of obstructive thrombi, which cause white matter lesions or lacunar infarcts [35]. In this context, all these lesions might be based on the fragility of vasculature irrespective of their different radiologic findings [36].

In addition, hypertension was significantly associated with the severity of CMBs. Large prospective cohort studies have also reported that hypertension is a risk factor for CMBs [25]. In particular, CMBs in the corticosubcortical junction or infratentorial lesions result from hypertensive or atherosclerotic microangiopathy. Hypertensive burden strongly affect the microvasculature of both the brain and kidneys [37]. In support of this, modulators of the renin angiotensin system have been shown to be effective in preventing proteinuria and also the aggravation of cerebral small vessel disease [38,39]. Optimal blood pressure control is regarded as the foremost determinant of cerebrovascular and renal protection [40].

The estimated GFR failed to show an association after adjusting for the white matter lesions. The white matter lesion volume may play a role as a potent confounder for CMBs in this study [41]. Microalbuminuria was not associated with CMBs in our results. A previous study reported that patients with a proteinuria grade of one or more had at least a twofold increased risk of having CMBs compared to patients with trace proteinuria or none at all [29]. This difference might be explained by age and different ethnicities which may affect the urine albumin concentrations. Our group was relatively younger than those of the previous study, and different levels of albumin secretion according to ethnicity may have contributed to the small proportion of patients with overt proteinuria [42].

The association between cystatin C levels and CMBs could be understood by the manifestation of microvascular damage of two end organs, the brain and the kidneys. Both vascular beds are exposed to high pulsatile pressure on account of upstream vasodilation. They are passively perfused at a high flow rate throughout systolic and diastolic periods, and by contrast, their smallest arteries have low resistance [7,19,37]. Thus, they are exposed to high shear stress, and are susceptible to hypertensive insults and variations in blood pressure [43]. CMBs are indicators of previous occurrences of blood extravasation from advanced fibro-hyalinized arterioles. Chronic kidney disease is also characterized by glomerular endothelial dysfunction affected by lipohyalinosis [44,45]. In addition, nitric oxide plays an important role in the proliferation of smooth muscle and maintenance of constant blood flow. Decreased nitric oxide levels in the degenerated endothelium of microangiopathic vessels was found to increase pro-inflammatory and pro-thrombotic properties, and eventually lead to a loss of blood flow auto-regulation [46]. Finally, sodium retention, activation of the renin-angiotensin system, and elevated catecholamine levels lead to increased blood pressure [47]. These effects might partially contribute to the occurrence of CMBs in renal insufficiency. Considering the similar pathomechanisms in small vessel diseases of the brain and kidney, cystatin C, a more reliable marker of renal function, might represent the degree of severity of CMBs more accurately [17].

Growing evidence suggested that CMBs are an indicator of a bleeding prone brain. CMBs are closely associated with the occurrence and outcome of hemorrhagic transformation after thrombolysis in ischemic stroke [48]. Their location and numbers are potent predictors for the outcome of spontaneous or antithrombotic related intracranial hemorrhages [3,49]. The association between renal indicators and CMBs may explain in part why these trends were more profound in patients with chronic kidney disease [50].

There are some caveats to this study. First, this is a cross-sectional study and no causal relationship between CMBs and cystatin C can be evaluated. Second, cystatin C levels were not measured repeatedly. An acute phase reaction accompanying stroke or the stroke severity may influence the levels of cystatin C, though information about the changes in cystatin C levels after acute cardiovascular events has not been reported. Third, this study was conducted in patients with stroke. The included parties probably had greater co-morbidities, e.g. diabetes and hypertension, which are also risk factors for CMBs, than a community based population; thus, they were likely to have more CMBs than a healthy population. These results should be confirmed in a healthy population with various ethnicities. Fourth, we used GRE scans at diagnosis of stroke. The presence of CMBs might be affected by the ischemic stroke [51]. However, the rapid appearance of CMBs after stroke was only related to the baseline number of CMBs and white matter lesion volumes. Thus, this bias had little impact on our results. Fifth, we were unable to obtain a history of the previous use of cardiovascular medication in some patients meticulously. This might be one confounder in our study as a recent study has reported the association between these medications and CMBs [52].

## Conclusions

Our results showed that cystatin C may be a more sensitive indicator to detect the severity of CMBs compared to the estimated GFR or microalbumin/creatinine ratio in patients with ischemic stroke. CMBs strongly correlated with the occurrence and clinical outcome of intracranial hemorrhages. Given its association with the severity of CMBs, cystatin C levels could help stratify the risk for intracranial hemorrhage more accurately. Further studies are needed to clarify this issue.

## Competing interests

The authors declare that they have no competing interests.

## Authors’ contributions

M-YO, BWY, S-HL, JSK, and HL contributed to conception and design, or acquisition of data, or analysis and interpretation of data. M-YO, HL, SBK, W-SR, CK, and CHK involved in drafting the manuscript or revising it. All authors read and approved the final manuscript.

## Pre-publication history

The pre-publication history for this paper can be accessed here:

http://www.biomedcentral.com/1471-2377/14/127/prepub

## Supplementary Material

Additional file 1: Table S1 Proportional ordinal logistic regression for the grades of CMBs using the clinical categories of estimated GFR and cystatin C quartiles. Click here for file

Additional file 2: Table S2 Proportional ordinal logistic regression for the grades of CMBs without the patients with renal failure. Click here for file

Additional file 3: Table S3 Proportional ordinal logistic regression for the grades of CMBs using estimated GFR based on creatinine and cystatin C. Click here for file

Additional file 4: Table S4 Proportional ordinal logistic regression for the grades of CMBs without the patients with renal failure. Click here for file

Additional file 5: Table S5 Proportional odds logistic regression analyses using quartiles of cystatin C and estimated GFR in the group with deep or infratentorial CMBs. Click here for file

Additional file 6: Table S6 Proportional odds logistic regression analyses using quartiles of cystatin C and albumin/creatinine in the group with deep or infratentorial CMBs. Click here for file
