# Supplementary material for: Cystatin C, a novel indicator of renal function, reflects severity of cerebral microbleeds
Source: BMC Neurol. 2014 Jun 12;14:127. doi: 10.1186/1471-2377-14-127 (PMC4077563; doi:10.1186/1471-2377-14-127)
Supplement: Additional file 1: Table S1 — Proportional ordinal logistic regression for the grades of CMBs using the clinical categories of estimated GFR and cystatin C quartiles. [file 1471-2377-14-127-S1.pdf]

Supplemental table1, proportional ordinal logistic regression for the grades of CMBs using the categories of estimated GFR and cystatin C quartiles.

Variables	N	unadjusted OR	95% CI	*p*	adjusted OR	95% CI	*p*
Categories of estimated GFR,ml/min/1.73m^2							
C4(≤15.0)	13	1.38	0.45-4.23	0.30	1.68	0.19-1.87	0.38
C3(15.0-45.0)	33	1.25	0.75-2.08	0.17	1.86	0.26-1.10	0.09
C2(45.0-60.0)	70	2.24	1.17-4.31	<0.01	1.41	0.41-1.22	0.22
C1(≥60.0),ref	567						
p for trend				<0.01			0.06
Quartiles of Cystatin C, nmol/L							
Q4(≥66.7)	168	2.19	1.40-3.40	<0.01	2.03	1.19-3.47	<0.01
Q3(54.7-66.7)	173	1.57	0.92-2.46	0.10	1.34	0.44-1.26	0.27
Q2(47.2-54.7)	162	0.98	0.61-1.58	0.16	1.59	0.91-2.79	0.11
Q1(≤47.2), ref	180						
p for trend				<0.01			<0.01

* adjusted for covariates; age, sex, total cholesterol, diabetes, hypertension, dyslipidemia, previous heart disease, smoking, previous anti thrombotic or anticoagulant use, and white matter lesions
