# Supplementary material for: Cystatin C, a novel indicator of renal function, reflects severity of cerebral microbleeds
Source: BMC Neurol. 2014 Jun 12;14:127. doi: 10.1186/1471-2377-14-127 (PMC4077563; doi:10.1186/1471-2377-14-127)
Supplement: Additional file 2: Table S2 — Proportional ordinal logistic regression for the grades of CMBs without the patients with renal failure. [file 1471-2377-14-127-S2.pdf]

Supplemental table2. proportional ordinal logistic regression for the grades of CMBs without the patients with renal failure. .

Variables	N	unadjusted OR	95% CI	*p*	adjusted OR	95% CI	*p*
Categories of estimated GFR,ml/min/1.73m^2							
Q4(≤66.1)	158	2.01	1.75-5.07	<0.01	1.28	0.23-2.66	0.16
Q3(66.1-78.8)	175	1.25	0.81-2.34	0.27	0.78	0.59-2.83	0.40
Q2(78.8-94.7)	164	0.77	0.77-2.21	0.32	1.11	0.49-1.64	0.31
Q1(≥94.7),ref	173						
p for trend				<0.01			0.35
Quartiles of Cystatin C, nmol/L							
Q4(≥66.7)	156	2.40	1.40-3.40	<0.01	1.88	1.05-3.38	0.03
Q3(54.7-66.7)	172	1.85	1.10-3.13	0.02	1.24	0.74-2.08	0.26
Q2(47.2-54.7)	162	1.45	0.61-1.58	0.16	1.21	0.49-1.41	0.27
Q1(≤47.2), ref	180						
p for trend				<0.01			0.03

* adjusted for covariates; age, sex, total cholesterol, diabetes, hypertension, dyslipidemia, previous heart disease, smoking, previous anti thrombotic or anticoagulant use, and white matter lesions
