# Supplementary material for: Cystatin C, a novel indicator of renal function, reflects severity of cerebral microbleeds
Source: BMC Neurol. 2014 Jun 12;14:127. doi: 10.1186/1471-2377-14-127 (PMC4077563; doi:10.1186/1471-2377-14-127)
Supplement: Additional file 3: Table S3 — Proportional ordinal logistic regression for the grades of CMBs using estimated GFR based on creatinine and cystatin C. [file 1471-2377-14-127-S3.pdf]

Supplemental table 3. proportional ordinal logistic regression for the grades of CMBs using estimated GFR based on creatinine and cystatin C

Variables	unadjusted OR	95% CI	*p*	adjusted OR	95% CI	*p*
Estimated GFR based on creatinine and cystatin C, per SD	1.29	1.01-1.17	<0.01	1.25	1.02-1.15	0.03
Estimated GFR based on cystatin C alone, per SD	1.33	1.04-1.21	<0.01	1.28	1.02-1.12	0.03
Estimated GFR based on creatinine alone, per SD	1.23	0.95-1.10	0.46	1.22	0.94-1.09	0.80

* GFR: glomerular filtration rate, SD: standard deviation

†adjusted for covariates; age, sex, total cholesterol, diabetes, hypertension, dyslipidemia, previous heart disease, smoking, previous anti thrombotic or anticoagulant use, and white matter lesions
