# Supplementary material for: Cystatin C, a novel indicator of renal function, reflects severity of cerebral microbleeds
Source: BMC Neurol. 2014 Jun 12;14:127. doi: 10.1186/1471-2377-14-127 (PMC4077563; doi:10.1186/1471-2377-14-127)
Supplement: Additional file 4: Table S4 — Proportional ordinal logistic regression for the grades of CMBs without the patients with renal failure. [file 1471-2377-14-127-S4.pdf]

Supplemental table 4. proportional ordinal logistic regression for the grades of CMBs without the patients with renal failure

Variables	N	unadjusted OR	95% CI	*p*	adjusted OR	95% CI	*p*
Quartiles of Cystatin C, nmol/L							
Q4(≥66.7)	156	2.40	1.40-3.40	<0.01	2.06	1.07-3.94	<0.01
Q3(54.7-66.7)	172	1.85	1.10-3.13	0.02	2.20	1.18-4.09	0.01
Q2(47.2-54.7)	162	1.45	0.61-1.58	0.16	1.36	0.78-2.36	0.49
Q1(≤47.2), ref	180						
p for trend				<0.01			<0.01
Quartiles of albumin/creatinine,							
Q4(≥0.10)	150	1.39	0.77-2.53	0.28	1.34	0.71-1.43	0.30
Q3(0.02-.0.10)	133	1.21	0.67-2.20	0.30	1.28	0.73-1.48	0.25
Q2(0.01-0.02)	217	1.25	0.40-1.62	0.36	0.86	0.53-1.61	0.64
Q1(≤0.01),ref	170						
p for trend				0.10			0.25

* adjusted for covariates; age, sex, total cholesterol, diabetes, hypertension, dyslipidemia, previous heart disease, smoking, previous anti thrombotic or anticoagulant use, and white matter lesions
