# Supplementary material for: Cystatin C, a novel indicator of renal function, reflects severity of cerebral microbleeds
Source: BMC Neurol. 2014 Jun 12;14:127. doi: 10.1186/1471-2377-14-127 (PMC4077563; doi:10.1186/1471-2377-14-127)
Supplement: Additional file 5: Table S5 — Proportional odds logistic regression analyses using quartiles of cystatin C and estimated GFR in the group with deep or infratentorial CMBs. [file 1471-2377-14-127-S5.pdf]

Supplemental table 5. Proportional odds logistic regression analyses using quartiles of cystatin C and estimated GFR in the group with deep or infratentorial CMBs

Variables	N	unadjusted OR	95% CI	*p*	adjusted OR	95% CI	*p*
Quartiles of Cystatin C							
Q4(≥66.7)	45	5.00	1.30-1.91	0.02	5.63	1.01-31.67	0.04
Q3(54.7-66.7)	39	1.50	0.51-4.34	0.46	1.35	0.30-5.91	0.69
Q2(47.2-54.7)	23	1.00	0.40-2.28	0.93	1.01	0.31-3.32	0.97
Q1(≤47.2), ref	28						
p for trend				0.02			0.04
Quartiles of estimated GFR							
Q4(≤66.1)	5	1.16	0.13-10.14	0.90	1.18	0.10-14.35	0.89
Q3(66.1-78.8)	11	1.15	0.29-4.46	0.83	0.43	0.10-2.70	0.37
Q2(78.8-94.7)	16	0.78	0.26-2.27	0.65	0.77	0.17-3.49	0.74
Q1(≥94.7),ref	103						
p for trend				0.95			0.67

*adjusted for the covariates:age, sex, total cholesterol, diabetes, hypertension, dyslipidemia, previous heart disease, smoking, previous anti thrombotic or anticoagulant use and white matter lesions
