# Supplementary material for: Cystatin C, a novel indicator of renal function, reflects severity of cerebral microbleeds
Source: BMC Neurol. 2014 Jun 12;14:127. doi: 10.1186/1471-2377-14-127 (PMC4077563; doi:10.1186/1471-2377-14-127)
Supplement: Additional file 6: Table S6 — Proportional odds logistic regression analyses using quartiles of cystatin C and albumin/creatinine in the group with deep or infratentorial CMBs. [file 1471-2377-14-127-S6.pdf]

Supplemental table 6. Proportional odds logistic regression analyses using quartiles of cystatin C and albumin/creatinine in the group with deep or infratentorial CMBs

Variables	N	unadjusted OR	95% CI	*p*	adjusted OR	95% CI	*p*
Quartiles of Cystatin C							
Q4(≥66.7)	45	5.00	1.30-1.91	0.02	7.00	1.24-35.46	0.03
Q3(54.7-66.7)	39	1.50	0.51-4.34	0.46	2.95	0.31-2.76	0.42
Q2(47.2-54.7)	23	1.00	0.40-2.28	0.93	2.81	0.52-1.49	0.23
Q1(≤47.2), ref	28						
p for trend				0.02			0.02
Quartiles of albumin/creatinine							
Q4(≥0.10)	41	1.40	0.39-5.00	0.61	3.60	0.71-28.00	0.22
Q3(0.02-.0.10)	41	1.00	0.26-3.08	0.87	1.58	0.73-1.15	0.65
Q2(0.01-0.02)	25	1.23	0.30-5.03	0.77	2.00	0.20-1.96	0.55
Q1(≤0.01),ref	28						
p for trend				0.72			0.26

*adjusted for the covariates:age, sex, total cholesterol, diabetes, hypertension, dyslipidemia, previous heart disease, smoking, previous anti thrombotic or anticoagulant use and white matter lesions
